# Author Correction: Glassy Dynamics in a heavy ion irradiated NbSe_2_ crystal

**DOI:** 10.1038/s41598-018-32999-7

**Published:** 2018-09-26

**Authors:** S. Eley, K. Khilstrom, R. Fotovat, Z. L. Xiao, A. Chen, D. Chen, M. Leroux, U. Welp, W. K. Kwok, L. Civale

**Affiliations:** 1 Condensed Matter and Magnet Science, MPA, Los Alamos National Laboratory, Los Alamos, NM 87545 USA; 2 Department of Physics, Colorado School of Mines, Golden, CO 80401 USA; 3 Materials Science Division, Argonne National Laboratory, Argonne, IL 60439 USA; 4 Center for Integrated Nanotechnology (CINT), MPA, Los Alamos National Laboratory, Los Alamos, NM 87545 USA; 5 Materials Science and Technology Division, MST-8, Los Alamos National Laboratory, Los Alamos, NM 87545 USA

Correction to: *Scientific Reports* 10.1038/s41598-018-31203-0, published online 03 September 2018

In the original version of this Article, the ORCID ID for S. Eley was omitted.

In addition, the original version of Figure 1 was incorrectly labelled as Figure 1a.

These errors have now been corrected in the HTML and PDF versions of the Article.

